# First Detection of Benign Rabbit Caliciviruses in Chile

**DOI:** 10.3390/v16030439

**Published:** 2024-03-12

**Authors:** Elena Smertina, Luca M. Keller, Nina Huang, Gabriela Flores-Benner, Jennifer Paola Correa-Cuadros, Melanie Duclos, Fabian M. Jaksic, Cristóbal Briceño, Victor Neira Ramirez, Miguel Díaz-Gacitúa, Sebastián Carrasco-Fernández, Ina L. Smith, Tanja Strive, Maria Jenckel

**Affiliations:** 1 Commonwealth Scientific and Industrial Research Organisation, Health and Biosecurity, Black Mountain, Canberra, ACT 2601, Australia; elena.smertina@csiro.au (E.S.); lucakeller@yahoo.de (L.M.K.); nina.huang@csiro.au (N.H.); ina.smith@csiro.au (I.L.S.); tanja.strive@csiro.au (T.S.); 2 Departamento de Ecología, Facultad de Ciencias Biológicas, Pontificia Universidad Católica de Chile, Santiago 8331150, Chile; gvflores@bio.puc.cl (G.F.-B.); jpcorrea4@uc.cl (J.P.C.-C.); fjaksic@bio.puc.cl (F.M.J.); 3 Center of Applied Ecology and Sustainability (CAPES), Santiago 8331150, Chile; mduclos@bio.puc.cl (M.D.); sebastian.ignacio.c.f@gmail.com (S.C.-F.); 4 Centro de Investigación para la Sustentabilidad, Universidad Andrés Bello (CIS-UNAB), Santiago 8370251, Chile; 5 Departamento de Medicina Preventiva Animal, Facultad de Ciencias Veterinarias y Pecuarias, Universidad de Chile, Santiago 8330111, Chile; cristobal.briceno@uchile.cl (C.B.); victorneira@u.uchile.cl (V.N.R.); 6 Corporación Nacional Forestal (CONAF), Santiago 8330407, Chile; miguel.diaz@conaf.cl; 7 Magíster en Recursos Naturales, Facultad de Ciencias de la Vida, Universidad Andrés Bello, República 440, Santiago 8370251, Chile

**Keywords:** European rabbit, *Oryctolagus cuniculus*, invasive species, RCV, RHDV, viral pathogens

## Abstract

Pathogenic lagoviruses (Rabbit hemorrhagic disease virus, RHDV) are widely spread across the world and are used in Australia and New Zealand to control populations of feral European rabbits. The spread of the non-pathogenic lagoviruses, e.g., rabbit calicivirus (RCV), is less well studied as the infection results in no clinical signs. Nonetheless, RCV has important implications for the spread of RHDV and rabbit biocontrol as it can provide varying levels of cross-protection against fatal infection with pathogenic lagoviruses. In Chile, where European rabbits are also an introduced species, myxoma virus was used for localised biocontrol of rabbits in the 1950s. To date, there have been no studies investigating the presence of lagoviruses in the Chilean feral rabbit population. In this study, liver and duodenum rabbit samples from central Chile were tested for the presence of lagoviruses and positive samples were subject to whole RNA sequencing and subsequent data analysis. Phylogenetic analysis revealed a novel RCV variant in duodenal samples that likely originated from European RCVs. Sequencing analysis also detected the presence of a rabbit astrovirus in one of the lagovirus-positive samples.

## 1. Introduction

Rabbit calicivirus (RCV) is a non-pathogenic member of the *Lagovirus* genus in the Caliciviridae family that infects European rabbits (*Oryctolagus cuniculus*). RCV targets the small intestine, and the course of infection is largely asymptomatic with no apparent clinical signs [1,2,3]. By contrast, closely related pathogenic lagoviruses, such as the Rabbit haemorrhagic disease virus (RHDV)*,* cause fast-developing acute liver necrosis and haemorrhages with a high case fatality rate of >90% and have been detected worldwide [4]. The first RCV was detected in a rabbitry in Italy in the mid-1990s [1]. Another RCV was isolated from a healthy domestic rabbit in France in 2006 (both referred to as RCV-Europe 1, or RCV-E1) [5,6]. Subsequently, a different RCV strain was isolated in Australia (RCV-A1) [2]. RCV has since been found widely across both Australia and New Zealand, prevailing in temperate climate zones [7,8]. However, the spread of RCV across the world is largely unknown.

Both pathogenic and non-pathogenic rabbit caliciviruses share the same genome structure. The virus particles contain two types of RNA molecules: genomic RNA, about 7.5 kb that encodes both non-structural (e.g., helicase, protease, RNA-dependent RNA polymerase (RdRp)) and structural (capsid) proteins; and sub-genomic RNA, of around 2 kb encoding only the capsid proteins VP60 and VP10 [9,10,11]. VP60 is the major structural protein that forms the capsid and VP10 is involved in the viral genome release at the early stages of infection [12,13]. Both genomic and sub-genomic RNAs are covalently bound to the viral protein g (VPg) at the 5′-end and are polyadenylated at the 3′-end [11,14,15].

Lagoviruses are classified into genogroups (GI, GII), genotypes (GI.1, GI.2, etc.), and variants (GI.1a, GI.1b, etc.) [16]. For example, non-pathogenic RCV-A1 and RCV-E1 are referred to as GI.4 and GI.3 genotypes, respectively. RCVs of the GI.4 genotype are further divided into variants GI.4a–e [16]. Pathogenic RHDVs belong to GI.1 and GI.2 genotypes. Recombination events usually occur at the highly conserved breakpoint between the RdRp and the VP60 gene [17]. Based on the nomenclature, recombinants are named by the type of RdRp, followed by the type of VP60. For example, GI.4cP-GI.2 refers to a recombinant variant consisting of the non-structural genes of a non-pathogenetic GI.4c variant and the structural genes of the GI.2 genotype. Several pathogenic recombinants that contain the non-structural genes of RCV strains and the VP60 sequence of pathogenic lagoviruses have been described in the past [18,19,20].

Both RCV and RHDV are highly species-specific and only infect animals of the family *Leporidae* [21]. Therefore, these viruses have been used in pest management in Australia and New Zealand to reduce the populations of feral European rabbits—one of the most devastating vertebrate pest species in these countries [4,22]. Although this biocontrol strategy has drastically decreased the rabbit population size in both countries, there are a few factors that interfere with its efficiency, including the reported ability of benign RCV to provide a varied extent of immunological protection against pathogenic RHDV and therefore acting as an imperfect natural vaccine [1,3,22]. It has been observed that the level of protection depends on the time between RCV and RHDV infections and is highest if the infections occurred closely together [23,24]. At the same time, co-infection of RCV and RHDV can give rise to recombination variants with increased epidemiological fitness [17].

**Figure 1:**
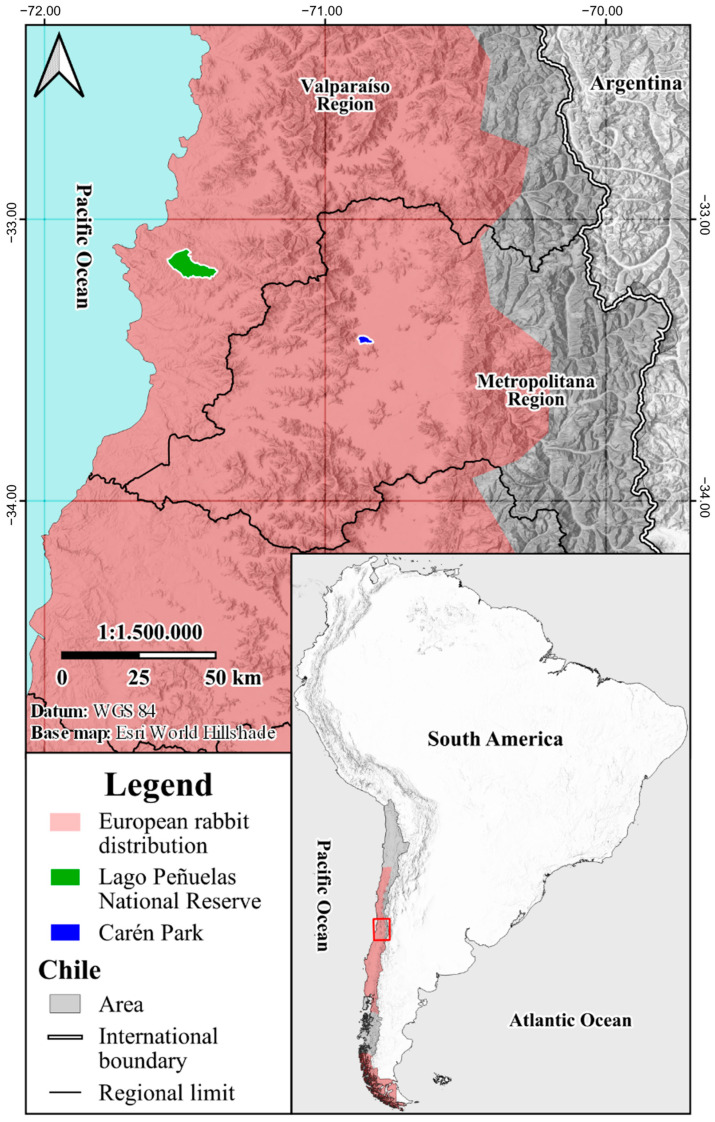
Distribution of the European rabbit (*Oryctolagus cuniculus*) in Chile (pink in inset) and location of sampling sites (red square in inset): Lago Peñuelas National Reserve (RNLP) (green) and Carén Park (CP) (blue).

The European rabbit is also an introduced species in Chile, with populations established by the late 18th century [25,26]. Rabbits are now widely distributed across the country (Figure 1) from the north (Atacama region) to the austral zone (Magallanes region), excluding the Aysén region, where there are apparently no stable populations, and some islands [26,27]. Feral rabbits cause significant damage to both the local ecosystems and economy by excessive grazing on native plant species and agricultural and forestry crops, producing soil erosion, facilitating the spread of introduced weeds, and competing for resources with farmed animals and native species [27,28,29].

In the 1950s, the serious impact of the rabbits on livestock in Isla Grande de Tierra del Fuego (a southernmost island shared by Chile and Argentina), led the Chilean government to release myxoma virus as a biological control agent [27]. This virus causes myxomatosis, a fatal disease in European rabbits which is characterised by major cutaneous lesions and death within two weeks following infection [30]. This localised biocontrol measure was highly effective, and rabbits were no longer a problem for the local livestock industries [26,31], although small populations remained at the southern end of the island [27,29]. From the 2010s, this virus began to be detected in central and southern Chile (between Coquimbo and Biobío regions) [27].

Myxoma virus was also released in Australia in the 1950s and, initially, rabbit numbers dropped dramatically following its introduction [4,27]. However, this was followed by a gradual recovery of the population size due to the emergence of less virulent myxoma virus strains and development of genetic resistance in rabbits [32,33]. To curb increasing rabbit numbers resulting from these co-evolutionary processes, Australia also imported the pathogenic lagovirus RHDV in the 1990s, to maintain the benefits of rabbit biocontrol [34]. A later study determined the economic benefits of the various cumulative rabbit biocontrol initiatives to exceed AUD 70 billion between 1950 and 2011 [35]. In 2015, a second pathogenic RHDV (RHDV2) that was not deliberately released arrived in Australia and resulted in a reduction of wild rabbit populations by 60% on average [36,37], maintaining some of the long-term benefits of lagovirus-mediated rabbit biocontrol.

In Chile, it is mandatory to report rabbit haemorrhagic disease to the Agricultural and Livestock Service (SAG) because it is classified as an exotic pathogen, and its use as a biological control agent is prohibited [27,38,39]. No data are available on the presence and/or spread of either pathogenic or non-pathogenic rabbit caliciviruses in Chile. In this work, we describe the first detection of naturally circulating RCV in Chile, which was identified in duodenum samples collected from rabbits in central Chile (Valparaíso and Metropolitan regions). In addition, a rabbit astrovirus was identified in one of the lagovirus-positive samples. Astroviruses are small RNA viruses (6.1–7.3 kb in length) that infect a wide range of mammalian and avian species targeting the gastro-intestinal tract, and in some cases cause gastroenteritis [40,41].

## 2. Methods

### 2.1. Sample Collection

Liver and duodenum samples were collected from both trapped and shot rabbits from two different sampling locations in central Chile: Lago Peñuelas National Reserve (RNLP) (9262 ha) located in the Valparaíso region, administrated by the National Forestry Corporation (CONAF) and Carén Park (CP) (1022 ha) located in the Metropolitan region, owned by the University of Chile and managed by Valle Lo Aguirre Foundation (Figure 1). Both locations belong to the Mediterranean biome, with a semiarid climate.

A systematic bimonthly sampling was conducted between October 2021 and June 2022 alternating between RNLP and CP. Captures of European rabbits were carried out with Tomahawk live traps and by hunting. The trap constitutes a metal cage with bait inside; once the bait is reached by an animal, the door locks. In the case of capture with live traps, the animals were euthanised under the standards defined by the American Veterinary Medical Association [42] by inhalation anaesthetic induction (sevoflurane) and subsequent cervical dislocation. All field activities were conducted with institutional permissions: hunting and capture license issued by the Servicio Agrícola y Ganadero (SAG, Resolution N° 1406/2021), permit for research within state protected wildlife areas (CONAF, N° 002/2021) and bioethics permit issued by Pontificia Universidad Católica de Chile (N° 210923003).

Rabbits were necropsied for tissue sampling: liver and duodenum samples (0.5 × 0.5 cm) were stored in 2 mL Eppendorf microtubes with RNAlater solution as stabiliser, labelled and delivered to the Animal Virology Laboratory of the Faculty of Veterinary and Livestock Sciences of the University of Chile, where they were stored frozen at −80 °C. Liver and duodenum samples were individually transported in leak-proof tubes containing DNA/RNA Shield (Zymo Research, Irvine, CA, USA) and shipped to CSIRO in Canberra, Australia, following national and international regulations under import permit number #0004801465 issued to CSIRO.

### 2.2. RNA Extraction and Virus Detection

Tissue (30 mg) was homogenised using glass beads and a Precellys tissue homogeniser (Bertin Technologies, Montigny-le-Bretonneux, France). Total RNA was extracted using the Maxwell^®^ RSC instrument (Promega, Madison, WI, USA) in combination with the Maxwell^®^ RSC SimplyRNA Tissue Kit (Promega, Madison, WI, USA). RNA of a total of 113 paired liver and duodenum samples (90 from RNLP and 23 from LC) were screened for the presence of rabbit caliciviruses using the broad-range SYBR-green-based RT-qPCR assay [43]. Samples positive for lagoviruses were subject to subsequent sequencing.

### 2.3. Sequencing and Data Analysis

A total of eight duodenum samples positive for rabbit caliciviruses were processed for total RNA sequencing. Libraries were prepared for metatranscriptomic sequencing, as previously described [44]. Briefly, total RNA was subject to an rRNA depletion step using the NEBNext rRNADepletion Kit (Human/Mouse/Rat) (New England Biolabs, Ipswich, MA, USA). rRNA-depleted RNA was subsequently prepared for sequencing using the NEB-Next Ultra II RNA Library Prep Kit for Illumina (New England Biolabs, Ipswich, Massachusetts, United States) according to manufacturer’s instructions. The quality of generated libraries was assessed using the Agilent 2200 TapeStation System and D1000 high sensitivity tapes (Agilent Technologies, Santa Clara, CA, USA). Sequencing was performed on a NovaSeq SP lane (300 cycles) at the Australian Genome Research Facility (AGRF) in Melbourne, Australia.

The quality of the sequenced data was assessed using FastQC (v0.11.08) [45]. Adapters were detected, trimmed, and paired reads were merged using fastp (v0.23.2) [46]. Reads that passed the quality control were then mapped to the *Oryctolagus cuniculus* reference genome (GCA_000003625.1) to remove the reads originating from the host. The remaining sequences were subject to de novo assembly using MEGAHIT (v1.2.9) [47]. The resulting contigs were then compared to the NCBI database using BLAST. Reads were mapped against contigs that were identified as viral using algorithms as implemented in Geneious Prime (v2023.1.2) and consensus sequences were generated.

### 2.4. Phylogenetic Analysis

A total of 76 non-structural and 101 structural RCV sequences, including the 8 newly identified sequences, were used to infer a phylogeny for non-structural and structural genes, respectively. The sequences were trimmed to remove the 3′ and 5′ untranslated regions (UTRs). MAFFT algorithm [48] accessed in Geneious Prime (v2023.2.1) was used to generate the alignments. The phylogenies were built using the maximum likelihood method in the IQ-TREE software (v2.2.0.5) [49] with the best fitting models SYM+I+G4 and GTR+F+I+G4 for the non-structural and structural RCV genes, respectively. Branch support was estimated using 1000 ultra-fast bootstrap replicates. The trees were rooted at the branch leading to the GI.3 clade.

For the rabbit astrovirus analysis, a total of 45 whole genome reference sequences of the *Astroviridae* family, available in the NCBI database, were used. The sequences were trimmed to remove the highly variable 5′ and 3′ UTRs. As described above, a MAFFT-generated alignment was used to build an unrooted phylogeny in IQ-TREE software with the best fitting model TVMe+I+G4 and branch support estimate using 1000 bootstrap replicates.

The phylogenetic trees were visualised and annotated in R [50] using packages ggtree (v3.8.0) [51], treeio (v1.24.1) [52], dplyr (v1.1.2) [53], tidyverse (v2.0.0) [54], ggplot2 (v3.4.2) [55], svglite (v2.1.1) [56], RColorBrewer (v1.1-3) [57], ggnewscale (v0.4.9) [58], ggthemes (v4.2.4) [59], ggtreeExtra (v1.10.0) [60], plotly (v4.10.2) [61], data.table (v1.14.8) [62] and scales (v1.2.0) [63]. Inkscape was used for the astrovirus tree visualisation (https://inkscape.org; accessed on 8 November 2023).

### 2.5. Phylogenetic Molecular Clock Analysis

Available RCV sequences were aligned, and a phylogenetic tree was calculated as described above, and the temporal signal for the non-structural and structural sequences was assessed in TempEst [64]. The structural protein gene phylogeny was found to demonstrate sufficient temporal signal (correlation coefficient = 0.917) and was used for the subsequent analysis. The TMRCA of GI.4f variants was estimated using the Bayesian Markov chain Monte Carlo (MCMC) method, available in the Bayesian Evolutionary Analysis by Sampling Trees (BEAST) package v1.10.4 [65]. Marginal likelihood estimations, stepping stone and path sampling, as implemented in BEAST, were used to assess the most appropriate clock model (strict vs. uncorrelated relaxed clock) and population size (constant vs. exponential growth vs. Bayesian skyride). A strict clock together with a constant population size coalescent model was found to be most appropriate for the data as previously described [17]. Each analysis was run three times with 80,000,000 chains to convergence (ESS > 200). A maximum credibility tree was calculated with a 10% burn-in using TreeAnnotator (v1.10.4) as available in the package.

## 3. Results

### 3.1. Detection and Phylogenetic Analysis of Benign Rabbit Caliciviruses

Total extracted RNA from 113 liver and paired duodenum samples were screened for lagoviruses using a broad-range lagovirus RT-qPCR assay [43]. All liver samples tested negative. PCR products were obtained from eight duodenal samples with cycle threshold (Ct) values ranging from 33.21 to 39.21. The eight lagovirus-positive cases came from the Lago Peñuelas National Reserve (RNLP) (Figure 1) and were collected during austral spring (October to December 2021). These samples were subject to RNA sequencing.

**Figure 2:**
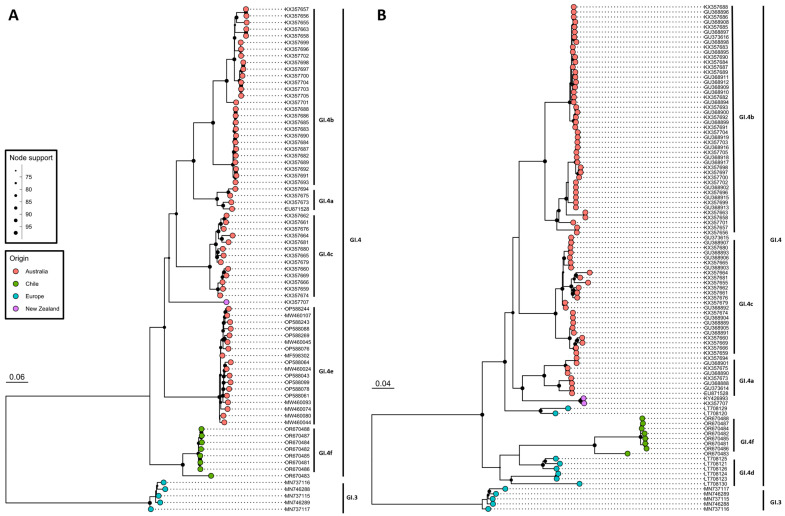
Maximum likelihood phylogenetic analysis of the newly identified RCV isolates from Chile (green dots). Non-structural (**A**) and capsid (**B**) RCV genes represented by 76 and 101 sequences, respectively, were used for the analysis. The phylogeny was generated with IQ-TREE software (v2.2.0.5) [49] using the best-fitted model (SYM+I+G4 and GTR+F+I+G4, respectively) with node support estimate from 1000 ultra-fast bootstrap replicates. Node support is indicated by the node size. Only node support values above 70 were considered as well supported and are displayed. The GenBank accession numbers are used as sequence names. The origin of samples is indicated by the tip colour. Genotypes and variants are indicated by clade labels.

Total RNA sequencing and subsequent data analysis led to the generation of eight complete or near complete RCV genome sequences with an average coverage ranging from 12 to 400 (Supplementary Table S1). The generated genome sequences show an overall nucleotide identity of 98–99.9% for seven out of eight samples, with the remaining sequence showing only ~90% identity, which suggests the presence of two RCV variants in Chile. The comparison of newly generated RCV sequences to the NCBI database revealed that the non-structural gene sequences share about 84% nucleotide identity with the Australian GI.4e variant (RCV-A1) (Figure 2A). The analysis of the structural genes (VP60) showed approximately 85% identity with GI.4d RCV variants originating from Europe (RCV-E2) (Figure 2B). Phylogenetic analyses of the structural and non-structural gene sequences show a clearly distinct clade compared to other lagovirus sequences from the GI.3 and GI.4 genotypes. The majority of identified RCVs, therefore, represent a novel GI.4f variant. Further information is needed to classify the remaining outlier sequence. The phylogenetic analysis did not show any evidence of recombination.

### 3.2. Time Scale Estimate of GI.4f Evolution

**Figure 3:**
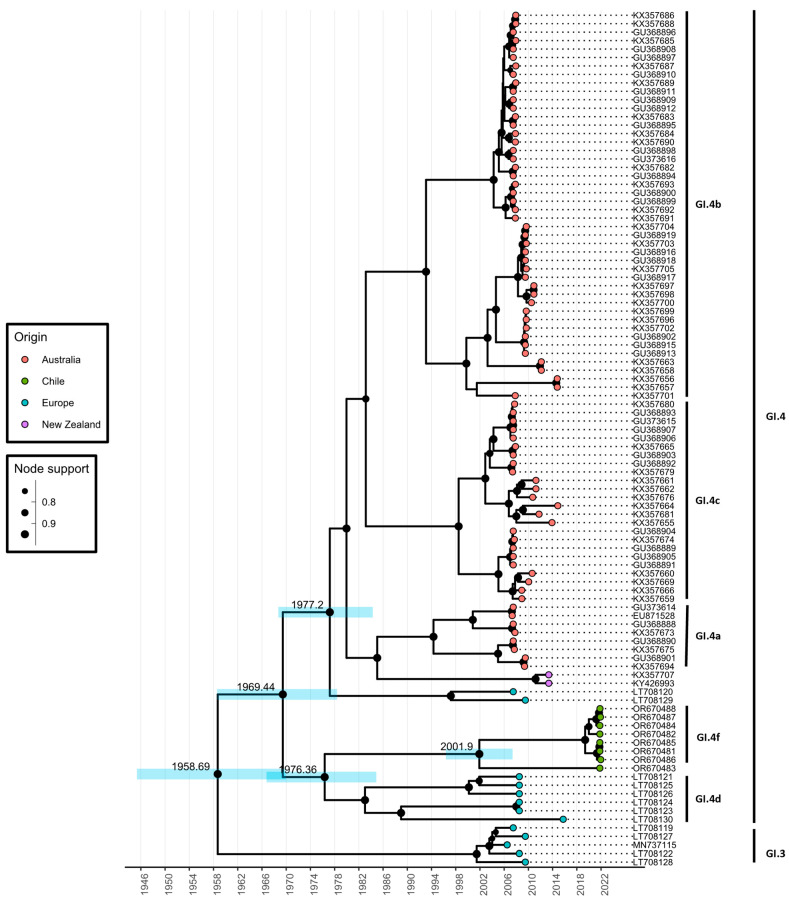
Evolutionary analysis of VP60 GI.4f isolates (green dots). The *x* axis represents a time scale in years. Median TMRCA values for GI.4f are shown at the nodes and blue node bars demonstrate 95% highest posterior density (HPD) intervals. Node support is indicated by the node size. Only node support values above 0.70 were considered as well supported and are displayed. The GenBank accession numbers are used as sequence names. Vertical lines on the right side indicate RCV variants and genotypes.

Evolutionary analysis using the MCMC approach was conducted with the RCV capsid gene sequences (VP60) described above to estimate the time range of Chilean RCV variants divergence from other known RCV variants and the time to most recent common ancestor (TMRCA) of GI.4f isolates. The analysis indicated that Chilean RCV shares a common ancestor with European variants (GI.4d) and diverged in the mid-1970s (median 1976.36; 95% HPD 1966.75–1984.88) suggesting that RCV had spread to South America from Europe (Figure 3). This timeframe overlaps with the calculated timeframe for the divergence of Australia/New Zealand RCV sequences from Europe (median 1977.2; 95% HPD 1968.71–1987.3). Furthermore, the analysis shows an additional separation of the Chilean clade into potentially two different variants that occurred between the late 1990s and early 2000s (median 2001.9; 95% HPD 1996.37–2007.39). However, more sequences are required to confirm this.

### 3.3. Coincidental Find of a Rabbit Astrovirus

**Figure 4:**
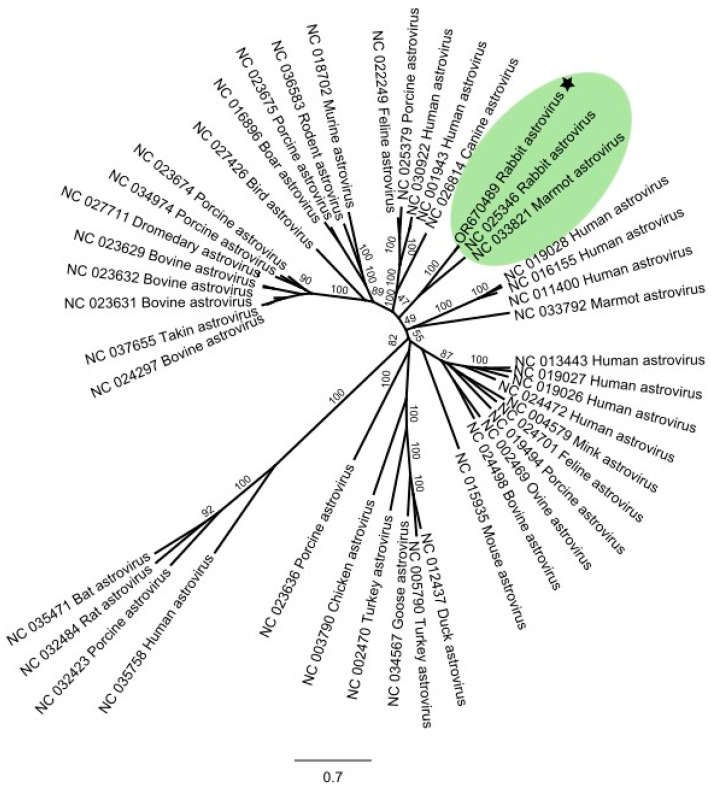
Unrooted phylogenetic tree of complete genome sequences of the family *Astroviridae*. The sequence identified in the duodenum sample from rabbits in Chile clusters with other rabbit and marmot astroviruses (highlighted in green) and is marked with a star. The GenBank accession number and host species from which the virus was isolated are used as sequence names. The scale bar represents the branch length corresponding to the number of substitutions between sequences. The phylogeny was generated using IQ-TREE software (v2.2.0.5) [49] with the best fitted model (TVMe+I+G4) and node support estimate from 1000 ultra-fast bootstrap replicates.

The sequence data analysis of one of the RCV positive duodenal samples also revealed the presence of an astrovirus in one of the samples. This sample came from RNLP, and the rabbit did not exhibit any clinical signs of infection. A whole genome with an average coverage of 340 was assembled (Supplementary Table S1). The phylogenetic analysis that included 45 published reference sequences for the family *Astroviridae* indicated that the astrovirus identified in the Chilean rabbit sample clusters with other known rabbit and marmot astroviruses (Figure 4). It is most closely related (91.6% nucleotide identity) to a known rabbit astrovirus (NC_025346) that was isolated from a domestic rabbit with gastroenteritis in the USA [66].

## 4. Discussion

To date, there has been no evidence of either pathogenic or non-pathogenic lagovirus circulation in Chile. In this study, a new variant of non-pathogenic RCV was identified in eight duodenum tissue samples from feral European rabbits in central Chile. All these samples originated from RNLP.

Based on the phylogenetic analysis of available non-structural and structural RCV gene sequences, the identified viruses represent at least one new variant, GI.4f, in line with the proposed nomenclature [16]. The non-structural genes are most closely related to the Australian GI.4e variant. For the capsid gene sequences, the GI.4d variant from Europe was identified as the closest cluster to the Chilean RCV isolates, thus positioning this variant between GI.4d and GI.4e variants. Of note, no non-structural gene sequences are available for the GI.4d variant, and the capsid gene sequences for GI.4e are missing from the analysis as well. While information on the non-structural genes for GI.4d is lacking, the GI.4e variant was identified as a recombinant only, with structural genes originating from GI.1 or GI.2 pathogenic RHDV genotypes [18,67]. Furthermore, the analysis shows that likely more than one RCV variant is circulating in Chile. However, only one sequence with a nucleotide difference of > 6% compared to the other Chilean sequences has been identified. Therefore, it cannot be classified as a separate variant [16].

According to the evolutionary analysis of the structural gene sequences, Chilean GI.4f and European GI.4d variants have a common ancestor and diverged around 1976; the same timeframe was estimated for the spread of RCV from Europe to Australia. The analysis, therefore, suggests that RCV likely arrived in Chile from Europe. However, the number of RCV sequences available for the analysis is limited, with most of the sequences originating from Australia, where active lagovirus surveillance has been carried in recent years due to the biocontrol measures involving pathogenic RHDV [68]. In other countries, the sampling may be limited, because samples from healthy rabbits are required due to the absence of clinical signs. With overall lack of sequence information for globally occurring RCV, the estimate for the arrival of RCV in Chile is rather uncertain and the timeframe for the divergence of Chilean and European RCV variants is quite broad, and more extensive sampling is necessary to narrow down the origin of Chilean RCV.

Although the rabbit population was established in both Australia and Chile around the late 18th century, the estimated divergence of Australian and European RCV isolates is similar to that for Chilean isolates, between 1970s and 1980s, with 1950s being the earliest estimate based on RHDV evolutionary rate which is lower than that of RCV [27,69]. Interestingly, the earliest estimate coincides with the introduction of myxoma virus to Australia and Chile [70,71], and it has been speculated that RCV could have been present in the imported virus preparations or rabbit materials [69]. It is also possible that earlier strains of Chilean RCV became locally extinct in areas that previously underwent sudden population bottlenecks caused by large myxoma outbreaks [27], leaving only more recent strains re-entering the recovering rabbit populations at a later stage to be sampled for this study. A previous study in Australia reported a decline in RCV prevalence in semi-arid environments following a sudden and drastic rabbit population reductions due to the arrival of pathogenic RHDV [7], rendering the likely density-dependent transmission of RCV more difficult and/or resulting in local extinctions of RCV. For a better understanding of RCV distribution in Chile, serological assays such as RCV-blocking ELISA could be utilised [72]. This assay detects RCV antibodies, allowing to conclude previous infections. The seroprevalence is also informative with regard to RHDV infections, as it is known that RCV can provide various levels of protection against pathogenic RHDV in RCV seropositive animals [3,23]. Further surveillance is required to determine the distribution of RCV across Chile.

An accidental finding from the sequence data analysis was the detection of a rabbit astrovirus. Although astroviruses can be associated with gastroenteritis, they are not believed to be the sole cause of the disease, as in some cases rabbits testing positive for astroviruses exhibit no clinical signs [66,73]. For the detected rabbit astrovirus, the association with disease in the sampled rabbit was not evident.

Studies on naturally circulating lagoviruses are important for several reasons. Firstly, genetic and evolutionary analysis of the rapidly evolving virus can provide information about the time of rabbit introductions and/or movements leading to the exposure of rabbit populations to these viruses [69]. Secondly, evolution from pre-existing non-pathogenic strains is believed to be one of the likely mechanisms leading to the emergence of virulent lagoviruses [74], warranting at least an opportunistic watching brief of potential reservoirs, i.e., areas where RCV is endemic. Finally, should virulent lagoviruses ever arrive in Chile, the presence of non-pathogenic lagoviruses may dampen their impact due to its ability to act as an imperfect vaccine [3].

## Data Availability

The RCV genomes sequenced in this study have been submitted to GenBank (accession numbers OR670481-OR670488). The rabbit astrovirus sequence is available under accession number OR670489.

## Supplementary Materials

The following supporting information can be downloaded at: https://www.mdpi.com/article/10.3390/v16030439/s1, Supplementary Table S1 contains summary of sequencing data pre and post quality control and merging paired reads; overview of mapping results to the *Oryctolagus cuniculus* reference genome (GCA_000003625.1); the identified RCV and rabbit astrovirus genomes.

## References

[1] Capucci L., Fusi P., Lavazza A., Pacciarini M.L., Rossi C. (1996). Detection and Preliminary Characterization of a New Rabbit Calicivirus Related to Rabbit Hemorrhagic Disease Virus but Nonpathogenic. J. Virol..

[2] Strive T., Wright J.D., Robinson A.J. (2009). Identification and Partial Characterisation of a New Lagovirus in Australian Wild Rabbits. Virology.

[3] Strive T., Wright J., Kovaliski J., Botti G., Capucci L. (2010). The Non-Pathogenic Australian Lagovirus RCV-A1 Causes a Prolonged Infection and Elicits Partial Cross-Protection to Rabbit Haemorrhagic Disease Virus. Virology.

[4] Kerr P.J., Hall R.N., Strive T. (2021). Viruses for Landscape-Scale Therapy: Biological Control of Rabbits in Australia. Methods Mol. Biol..

[5] Le Gall-Reculé G., Zwingelstein F., Fages M.P., Bertagnoli S., Gelfi J., Aubineau J., Roobrouck A., Botti G., Lavazza A., Marchandeau S. (2011). Characterisation of a Non-Pathogenic and Non-Protective Infectious Rabbit Lagovirus Related to RHDV. Virology.

[6] Lemaitre E., Zwingelstein F., Marchandeau S., Le Gall-Reculé G. (2018). First Complete Genome Sequence of a European Non-Pathogenic Rabbit Calicivirus (Lagovirus GI.3). Arch. Virol..

[7] Liu J., Fordham D.A., Cooke B.D., Cox T., Mutze G., Strive T. (2014). Distribution and Prevalence of the Australian Non-Pathogenic Rabbit Is Correlated with Rainfall and Temperature. PLoS ONE.

[8] Nicholson L.J., Mahar J.E., Strive T., Zheng T., Holmes E.C., Ward V.K., Duckworth J.A. (2017). Benign Rabbit Calicivirus in New Zealand. Appl. Environ. Microbiol..

[9] Ehresmann D.W., Schaffer F.L. (1977). RNA Synthesized in Calicivirus Infected Cells Is Atypical of Picornaviruses. J. Virol..

[10] Meyers G., Wirblich C., Thiel H.-J. (1991). Rabbit Hemorrhagic Disease Virus—Molecular Cloning and Nucleotide Sequencing of a Calicivirus Genome. Virology.

[11] Meyers G., Wirblich C., Thiel H.J. (1991). Genomic and Subgenomic RNAs of Rabbit Hemorrhagic Disease Virus Are Both Protein-Linked and Packaged into Particles. Virology.

[12] Parra F., Prieto M. (1990). Purification and Characterization of a Calicivirus as the Causative Agent of a Lethal Hemorrhagic Disease in Rabbits. J. Virol..

[13] Conley M.J., McElwee M., Azmi L., Gabrielsen M., Byron O., Goodfellow I.G., Bhella D. (2019). Calicivirus VP2 Forms a Portal-like Assembly Following Receptor Engagement. Nature.

[14] Black D.N., Burroughs J.N., Harris T.J.R., Brown F. (1978). The Structure and Replication of Calicivirus RNA. Nature.

[15] Burroughs J.N., Brown F. (1978). Presence of a Covalently Linked Protein on Calicivirus RNA. J. Gen. Virol..

[16] Le Pendu J., Abrantes J., Bertagnoli S., Guitton J.S., Le Gall-Reculé G., Lopes A.M., Marchandeau S., Alda F., Almeida T., Célio A.P. (2017). Proposal for a Unified Classification System and Nomenclature of Lagoviruses. J. Gen. Virol..

[17] Mahar J.E., Jenckel M., Huang N., Smertina E., Holmes E.C., Strive T., Hall R.N. (2021). Frequent Intergenotypic Recombination between the Non-Structural and Structural Genes Is a Major Driver of Epidemiological Fitness in Caliciviruses. Virus Evol..

[18] Lopes A.M., Dalton K.P., Magalhães M.J., Parra F., Esteves P.J., Holmes E.C., Abrantes J. (2015). Full Genomic Analysis of New Variant Rabbit Hemorrhagic Disease Virus Revealed Multiple Recombination Events. J. Gen. Virol..

[19] Abrantes J., Lopes A.M., Lemaitre E., Ahola H., Banihashem F., Droillard C., Marchandeau S., Esteves P.J., Neimanis A., Gall-Reculé G. (2020). Le Retrospective Analysis Shows That Most Rhdv Gi.1 Strains Circulating since the Late 1990s in France and Sweden Were Recombinant Gi.3p–Gi.1d Strains. Genes.

[20] Abrantes J., Droillard C., Lopes A.M., Lemaitre E., Lucas P., Blanchard Y., Marchandeau S., Esteves P.J., Le Gall-Reculé G. (2020). Recombination at the Emergence of the Pathogenic Rabbit Haemorrhagic Disease Virus Lagovirus Europaeus/GI.2. Sci. Rep..

[21] Urakova N., Hall R., Strive T., Frese M. (2019). Restricted Host Specificity of Rabbit Hemorrhagic Disease Virus Is Supported by Challenge Experiments in Immune-Compromised Mice (*Mus musculus*). J. Wildl. Dis..

[22] Cooke B.D., Duncan R.P., McDonald I., Liu J., Capucci L., Mutze G.J., Strive T. (2018). Prior Exposure to Non-Pathogenic Calicivirus RCV-A1 Reduces Both Infection Rate and Mortality from Rabbit Haemorrhagic Disease in a Population of Wild Rabbits in Australia. Transbound. Emerg. Dis..

[23] Strive T., Elsworth P., Liu J., Wright J.D., Kovaliski J., Capucci L. (2013). The Non-Pathogenic Australian Rabbit Calicivirus RCV-A1 Provides Temporal and Partial Cross Protection to Lethal Rabbit Haemorrhagic Disease Virus Infection Which Is Not Dependent on Antibody Titres. Vet. Res..

[24] Taggart P.L., Hall R.N., Cox T.E., Kovaliski J., McLeod S.R., Strive T. (2022). Changes in Virus Transmission Dynamics Following the Emergence of RHDV2 Shed Light on Its Competitive Advantage over Previously Circulating Variants. Transbound. Emerg. Dis..

[25] Camus P., Castro S., Jaksic F. (2008). European Rabbits in Chile: The History of a Biological Invasion. Hist. Santiago.

[26] Camus P., Castro S.A., Jaksic F.M. (2021). European Rabbit (*Oryctolagus cuniculus* L.) in Chile: The Human Dimension behind a Biological Invasion. Biological Invasions in the South American Anthropocene: Global Causes and Local Impacts.

[27] Correa-Cuadros J.P., Flores-Benner G., Muñoz-Rodríguez M.A., Briceño C., Díaz M., Strive T., Vásquez F., Jaksic F.M. (2023). History, Control, Epidemiology, Ecology, and Economy of the Invasion of European Rabbits in Chile: A Comparison with Australia. Biol. Invasions.

[28] Jaksic F.M. (1998). Vertebrate Invaders and Their Ecological Impacts in Chile. Biodivers. Conserv..

[29] PNUD Valoración Económica Del Impacto de Siete Especies Exóticas Invasoras Sobre Los Sectores Productivos y La Biodiversidad En Chile. https://especies-exoticas.mma.gob.cl/wp-content/uploads/2018/12/1.-LIBRO-Valoracion-economica-EEI-FINAL.pdf.

[30] Kerr P.J., Cattadori I.M., Rogers M.B., Fitch A., Geber A., Liu J., Sim D.G., Boag B., Eden J.S., Ghedin E. (2017). Genomic and Phenotypic Characterization of Myxoma Virus from Great Britain Reveals Multiple Evolutionary Pathways Distinct from Those in Australia. PLoS Pathog..

[31] Jaksic F.M., Yañez J.L. (1983). Rabbit and Fox Introductions in Tierra Del Fuego: History and Assessment of the Attempts at Biological Control of the Rabbit Infestation. Biol. Conserv..

[32] Kerr P.J., Best S.M. (1998). Myxoma Virus in Rabbits. Rev. Sci. Tech. Off. Int. Epiz.

[33] Alves J.M., Carneiro M., Cheng J.Y., Lemos De Matos A., Rahman M.M., Loog L., Campos P.F., Wales N., Eriksson A., Manica A. (2019). Parallel Adaptation of Rabbit Populations to Myxoma Virus. Science (1979).

[34] Cooke B.D., Fenner F. (2002). Rabbit Haemorrhagic Disease and the Biological Control of Wild Rabbits, *Oryctolagus cuniculus*, in Australia and New Zealand. Wildl. Res..

[35] Cooke B., Chudleigh P., Simpson S., Saunders G. (2013). The Economic Benefits of the Biological Control of Rabbits in Australia, 1950–2011. Aust. Econ Hist. Rev..

[36] Ramsey D.S., Patel K.K., Campbell S., Hall R.N., Taggart P.L., Strive T. (2023). Sustained Impact of RHDV2 on Wild Rabbit Populations across Australia Eight Years after Its Initial Detection. Viruses.

[37] Ramsey D.S.L., Cox T., Strive T., Forsyth D.M., Stuart I., Hall R., Elsworth P., Campbell S. (2020). Emerging RHDV2 Suppresses the Impact of Endemic and Novel Strains of RHDV on Wild Rabbit Populations. J. Appl. Ecol..

[38] Servicio Agrícola y Ganadero (SAG) Lista de enfermedades de denuncia obligatoria (EDO) al SAG. https://www.sag.gob.cl/sites/default/files/enfermedades_denuncia_obligatoria_sag_9-10-2019.pdf.

[39] Servicio Agrícola y Ganadero (SAG), Ord. No 274/2015.v1. Letter: Pronunciamiento Sobre La Aplicación de Calicivirus y Virus Mixoma Para Control de Conejo Europeo En Islas de Áreas Silvestres Protegidas Del Estado. Santiago, Chile, 2015. CSIRO Data Collection. https://data.csiro.au/collection/csiro%3A61523v1.

[40] Donato C., Vijaykrishna D. (2017). The Broad Host Range and Genetic Diversity of Mammalian and Avian Astroviruses. Viruses.

[41] Zhao Q., Tian Y., Liu L., Jiang Y., Sun H., Tan S., Huang B. (2022). The Genomic and Genetic Evolution Analysis of Rabbit Astrovirus. Vet. Sci..

[42] American Veterinary Medical Association (2020). AVMA Guidelines for the Euthanasia of Animals: 2020 Edition.

[43] Hall R.N., Mahar J.E., Read A.J., Mourant R., Piper M., Huang N., Strive T. (2018). A Strain-Specific Multiplex RT-PCR for Australian Rabbit Haemorrhagic Disease Viruses Uncovers a New Recombinant Virus Variant in Rabbits and Hares. Transbound. Emerg. Dis..

[44] Jenckel M., Hall R.N., Strive T. (2022). Pathogen Profiling of Australian Rabbits by Metatranscriptomic Sequencing. Transbound. Emerg. Dis..

[45] Andrews S. (2010). FastQC: A Quality Control Tool for High throughput Sequence Data [Online]. https://www.bioinformatics.babraham.ac.uk/projects/fastqc/.

[46] Chen S., Zhou Y., Chen Y., Gu J. (2018). Fastp: An Ultra-Fast All-in-One FASTQ Preprocessor. Bioinformatics.

[47] Li D., Liu C.M., Luo R., Sadakane K., Lam T.W. (2015). MEGAHIT: An Ultra-Fast Single-Node Solution for Large and Complex Metagenomics Assembly via Succinct de Bruijn Graph. Bioinformatics.

[48] Katoh K., Standley D.M. (2013). MAFFT Multiple Sequence Alignment Software Version 7: Improvements in Performance and Usability. Mol. Biol. Evol..

[49] Nguyen L.T., Schmidt H.A., Von Haeseler A., Minh B.Q. (2015). IQ-TREE: A Fast and Effective Stochastic Algorithm for Estimating Maximum-Likelihood Phylogenies. Mol. Biol. Evol..

[50] R Core Team (2023). R: A Language and Environment for Statistical Computing.

[51] Yu G., Smith D.K., Zhu H., Guan Y., Lam T.T.Y. (2017). Ggtree: An R Package for Visualization and Annotation of Phylogenetic Trees with Their Covariates and Other Associated Data. Methods Ecol. Evol..

[52] Wang L.G., Lam T.T.Y., Xu S., Dai Z., Zhou L., Feng T., Guo P., Dunn C.W., Jones B.R., Bradley T. (2020). Treeio: An R Package for Phylogenetic Tree Input and Output with Richly Annotated and Associated Data. Mol. Biol. Evol..

[53] Wickham H., François R., Henry L., Müller K., Vaughan D. (2023). Dplyr: A Grammar of Data Manipulation, R Package Version 1.1.2.

[54] Wickham H., Averick M., Bryan J., Chang W., McGowan L., François R., Grolemund G., Hayes A., Henry L., Hester J. (2019). Welcome to the Tidyverse. J. Open Source Softw..

[55] Wickham H. (2016). Ggplot2: Elegant Graphics for Data Analysis.

[56] Wickham H., Henry L., Pedersen T.L., Luciani T.J., Decorde M., Lise V. (2023). Svglite: An “SVG” Graphics Device, R Package Version 2.1.1.

[57] Neuwirth E. (2022). RColorBrewer: ColorBrewer Palettes.

[58] Campitelli E. (2023). Ggnewscale: Multiple Fill and Colour Scales in “Ggplot2”.

[59] Arnold J.B. (2023). Ggthemes: Extra Themes, Scales and Geoms for “Ggplot2”.

[60] Xu S., Dai Z., Guo P., Fu X., Liu S., Zhou L., Tang W., Feng T., Chen M., Zhan L. (2021). GgtreeExtra: Compact Visualization of Richly Annotated Phylogenetic Data. Mol. Biol. Evol..

[61] Sievert C. (2020). Interactive Web-Based Data Visualization with R, Plotly, and Shiny.

[62] Barrett T., Dowle M., Srinivasan A., Gorecki J., Chirico M., Hocking T. (2023). Data.Table: Extension of ‘data.Frame’. R Package Version 1.14.8.

[63] Wickham H., Pedersen T.L., Seidel D. (2022). Scales: Scale Functions for Visualization.

[64] Rambaut A., Lam T.T., Carvalho L.M., Pybus O.G. (2016). Exploring the Temporal Structure of Heterochronous Sequences Using TempEst (Formerly Path-O-Gen). Virus Evol..

[65] Drummond A.J., Rambaut A. (2007). BEAST: Bayesian Evolutionary Analysis by Sampling Trees. BMC Evol. Biol..

[66] Stenglein M.D., Velazquez E., Greenacre C., Wilkes R.P., Ruby J.G., Lankton J.S., Ganem D., Kennedy M.A., Derisi J.L. (2012). Complete Genome Sequence of an Astrovirus Identified in a Domestic Rabbit (*Oryctolagus cuniculus*) with Gastroenteritis. Virol. J..

[67] Mahar J.E., Read A.J., Gu X., Urakova N., Mourant R., Piper M., Haboury S., Holmes E.C., Strive T., Hall R.N. (2018). Detection and Circulation of a Novel Rabbit Hemorrhagic Disease Virus in Australia. Emerg. Infect. Dis..

[68] Peng N.Y.G., Hall R.N., Huang N., West P., Cox T.E., Mahar J.E., Mason H., Campbell S., O’Connor T., Read A.J. (2023). Utilizing Molecular Epidemiology and Citizen Science for the Surveillance of Lagoviruses in Australia. Viruses.

[69] Mahar J.E., Nicholson L., Eden J.-S., Duchêne S., Kerr P.J., Duckworth J., Ward V.K., Holmes E.C., Strive T. (2016). Benign Rabbit Caliciviruses Exhibit Evolutionary Dynamics Similar to Those of Their Virulent Relatives. J. Virol..

[70] Correa-Cuadros J.P., Flores-Benner G., María P.G., Ávila-Thieme I., Muñoz M., Duclos M., Soto N., Briceño C., Vásquez F., Díaz M. (2023). La Invasión del Conejo Europeo en Chile.

[71] Arentsen P. (1954). Control Biológico Del Conejo: Difusión Del Virus *Mixomatosis cuniculus*, Por Contagio Directo, En La Isla Grande de Tierra Del Fuego. Boletín Ganad. (Punta Arenas).

[72] Liu J., Kerr P.J., Strive T. (2012). A Sensitive and Specific Blocking ELISA for the Detection of Rabbit Calicivirus RCV-A1 Antibodies. Virol. J..

[73] Martella V., Moschidou P., Pinto P., Catella C., Desario C., Larocca V., Circella E., Bànyai K., Lavazza A., Magistrali C. (2011). Astroviruses in Rabbits. Emerg. Infect. Dis..

[74] Esteves P.J., Abrantes J., Bertagnoli S., Cavadini P., Gavier-Widén D., Guitton J.S., Lavazza A., Lemaitre E., Letty J., Lopes A.M. (2015). Emergence of Pathogenicity in Lagoviruses: Evolution from Pre-Existing Nonpathogenic Strains or through a Species Jump?. PLoS Pathog..

